# An Immune Gene Signature Stratifies Breast Cancer Prognosis Through iCAF-Driven Immunosuppressive Microenvironment

**DOI:** 10.3390/biomedicines13122966

**Published:** 2025-12-02

**Authors:** Sibin Mei, Chenhao Bai, Huijuan Wang, Kainan Lin, Tianyuan Pan, Yunkun Lu, Qian Cao

**Affiliations:** 1 Department of Gastroenterology, Sir Run-Run Shaw Hospital, Zhejiang University School of Medicine, Hangzhou 310016, China; meisibin@zju.edu.cn (S.M.);; 2 Department of Surgical Oncology, Sir Run-Run Shaw Hospital, Zhejiang University School of Medicine, Hangzhou 310016, China; 3 Department of General Surgery, Sir Run-Run Shaw Hospital, Zhejiang University School of Medicine, Hangzhou 310016, China; 4 Department of General Medicine, The First Affiliated Hospital of Zhejiang University School of Medicine, Hangzhou 310016, China

**Keywords:** breast cancer, prognostic signature, immune microenvironment, single-cell RNA sequencing, inflammatory CAFs

## Abstract

**Background/Objectives:** Breast cancer is the leading cause of cancer-related mortality in women, highlighting the urgent need for robust prognostic tools to enable individualized risk stratification. **Methods**: Transcriptomic data from 1075 breast cancer and 113 adjacent normal tissues in The Cancer Genome Atlas (TCGA) were integrated with clinical information. Differential expression analysis identified 531 immune-related genes, which were further selected by univariate Cox regression and Least Absolute Shrinkage and Selection Operator (LASSO) regression to construct a 13-gene prognostic signature. The model was validated in an independent cohort (*n* = 327). Tumor immune microenvironment and single-cell RNA sequencing data were analyzed to explore underlying biological differences. **Results**: The 13-gene signature effectively stratified patients into low- and high-risk groups with significantly different overall survival in both the TCGA cohort (log-rank *p* < 0.0001; C-index = 0.678; 5-year AUC = 0.72) and the validation cohort (log-rank *p* < 0.0001; C-index = 0.703; 3-year AUC = 0.81). Low-risk tumors exhibited an antitumor immune microenvironment enriched in CD8^+^ T cells, T follicular helper (Tfh) cells, and M1 macrophages, whereas high-risk tumors were dominated by immunosuppressive regulatory T cells and M2 macrophages (all *p* < 0.0001). Single-cell analysis revealed expansion of malignant epithelial cells and inflammatory cancer-associated fibroblasts (iCAFs) in high-risk tumors, with higher iCAF scores significantly associated with poorer survival (log-rank *p* = 0.00036). **Conclusions**: Collectively, this study delivers a rigorously validated 13-gene immune signature whose prognostic utility is rooted in distinct immune microenvironmental features, while unveiling iCAF-targeted therapeutic strategies as a promising intervention avenue.

## 1. Introduction

Breast cancer continues to be a leading cause of cancer-related mortality among women globally, with its complex pathophysiology posing significant challenges to effective diagnosis and treatment [1,2,3]. Breast cancer is characterized by heterogeneity with diverse clinical outcomes, underscoring the critical need for robust prognostic tools that can stratify patients into distinct risk categories [4,5,6]. Traditional clinicopathological factors often fail to capture the complexity of tumor biology, particularly the dynamic interplay between cancer cells and the immune microenvironment, which has emerged as a pivotal determinant of progression and therapeutic response. Recent research has highlighted the crucial role of the immune microenvironment in cancer development and progression [7,8,9,10,11,12,13,14]. For instance, combining anti-angiogenic agents with immune checkpoint blockade in neoadjuvant therapy can reshape the immune landscape, achieving high pathological complete response rates independent of PD-L1 status [11]. In lymph node-negative basal-like breast cancer, elevated tumor cell-intrinsic *CTLA4* expression paradoxically correlates with excellent prognosis by inducing T-cell exhaustion and establishing an immunosuppressive microenvironment that restricts tumor progression, challenging conventional views on checkpoint inhibitors as purely therapeutic targets [15]. An immune infiltration-derived prognostic score stratifies breast cancer survival by integrating heterogeneous immune cell abundances (e.g., TIL density, PD-1^+^ cells) and their dynamic crosstalk with genomic alterations, revealing microenvironment-driven immunoediting as a determinant of therapeutic vulnerability [16].

Recent advances in transcriptomic profiling have enabled the identification of immune-related gene signatures that reflect this interplay, offering promising avenues for precision oncology. For instance, multi-gene immune signatures have demonstrated prognostic utility in gastric cancer and ovarian cancer, highlighting their pan-cancer potential [17,18]. However, many existing signatures suffer from limitations such as inadequate validation, lack of biological interpretability, or failure to integrate microenvironmental context, necessitating more refined approaches. Current immune prognostic models often overlook the functional heterogeneity of tumor-infiltrating immune cells. While signatures derived from bulk RNA-seq data can stratify patients, they frequently lack resolution to dissect immunosuppressive mechanisms—such as the polarization of tumor-associated macrophages (M2-like) or infiltration of regulatory T cells (Tregs)—that drive aggressive phenotypes [19].

Recent studies highlight the dynamic role of cancer-associated fibroblasts (CAFs), particularly inflammatory CAFs (iCAFs), in sculpting immunosuppressive niches [19]. In pancreatic cancer, PPY-induced iCAFs recruit M2 macrophages and suppress CD8^+^ T cell function via EGFR/NF-κB signaling [20]. Similarly, in non-small cell lung cancer (NSCLC), iCAF enrichment predicts sensitivity to chemoimmunotherapy by enhancing Th17 responses and antigen-presenting cell interactions [21]. However, in breast cancer, the prognostic value of iCAFs and their integration into multimodal immune signatures remains underexplored. This gap is compounded by insufficient external validation across diverse cohorts, limiting clinical translation. The integration of single-cell technologies and large-scale real-world clinico-genomic data offers a path toward resolving these limitations, as evidenced by recent studies leveraging such approaches to identify predictive biomarkers across cancer types [22,23].

To bridge this gap, we leveraged transcriptomic data of 1075 breast tumors from The Cancer Genome Atlas (TCGA) to develop an immune-centric prognostic model. By intersecting differential expression analysis with Cox and Least Absolute Shrinkage and Selection Operator (LASSO) regression, we identified a 13-gene signature reflecting key immune mediators. This signature stratifies patients into distinct risk groups with divergent survival outcomes and immune microenvironments. Furthermore, single-cell RNA-seq revealed iCAFs as pivotal drivers of the high-risk phenotype—a finding validated across independent cohorts. Our study establishes a mechanistically anchored prognostic tool and nominates iCAF-targeted strategies as novel therapeutic avenues for aggressive breast cancer.

## 2. Materials and Methods

### 2.1. Data Collection and Identification of Immune-Related Genes

Transcriptomic data and clinical phenotypes of 1075 breast cancer samples and 113 adjacent normal tissues were obtained from the TCGA database as the training cohort (Table S1). For independent validation, RNA sequencing data and clinical information of 327 breast cancer samples were downloaded from the Gene Expression Omnibus (GEO) database [24] under accession GSE20685 (Table S2). Single-cell RNA sequencing data from six breast cancer samples were retrieved from GEO (accession GSE254991). Differential expression analysis between tumor and normal samples was performed using DESeq2 (v1.30.1) with adjacent normal tissues as controls [25]. Significantly differentially expressed genes (DEGs) were identified using thresholds of |log2 fold change| ≥ 1 and false discovery rate (FDR) ≤ 0.05. Functional enrichment analysis of DEGs was conducted using ClueGO (v2.5.10) with a significance threshold of *p* < 0.05 [26]. The resulting DEGs were intersected with 2407 immune-related genes curated from the ImmPort database [27] to identify breast cancer-associated immune-related DEGs.

### 2.2. Construction and Evaluation of the Immune-Related Gene Signature

Genes expressed (FPKM > 0) in fewer than 50% of samples were first excluded. Univariate Cox proportional hazards regression analysis was then performed using the “coxph” function from the “survival” R package (v3.8-3), retaining 63 genes with *p* ≤ 0.05. To refine the prognostic gene set and prevent overfitting, LASSO regression was implemented using the “cv.glmnet” function from the “glmnet” R package (v4.1-8), selecting the optimal lambda value through 10-fold cross-validation. This yielded a final 13-gene signature. The risk score for each patient was calculated as the sum of the product of each gene’s expression level and its corresponding LASSO-derived coefficient. Patients were stratified into high-risk and low-risk groups based on the median risk score. Gene expression patterns were visualized using the “pheatmap” package (v1.0.12). Kaplan–Meier survival analysis with log-rank testing was performed using the “survminer” package (v0.5.0). Time-dependent receiver operating characteristic (ROC) curves and area under the curve (AUC) values were generated using the “survivalROC” package (v1.0.3). Concordance indices (C-indices) were computed with the “survcomp” package (v1.56).

### 2.3. CIBERSORT Analysis and Statistical Methods

Immune cell infiltration profiles were estimated using the CIBERSORT-abs algorithm with the LM22 leukocyte signature matrix (containing 547 genes defining 22 immune cell subtypes) through the CIBERSORTx software from the website (https://cibersortx.stanford.edu/index.php, accessed on 28 March 2024) and the “CIBERSORT” R package (v1.04) [28]. Heatmaps depicting immune cell composition were generated using the “pheatmap” package (v1.0.12). Box plots comparing immune cell fractions between risk groups were created using “ggpubr” (v0.6.0) and “ggcorrplot” (v0.1.4). All statistical analyses were performed in R (v4.4.3). Continuous variables were compared using unpaired Student’s *t*-tests, with *p* < 0.05 considered statistically significant.

### 2.4. Single-Cell RNA Sequencing Data Analysis

The demultiplexing, barcoded processing, gene counting, and aggregation were made using the CeleScopesoftware (Singleron Biotechnologies, Nanjing, China; version v2.6.1). All scRNA-seq reads were aligned to the human reference genome (hg38), and the cell-by-gene count matrices were produced. The scRNA-seq expression profiles were analyzed using the R package Seurat (v4.0.4) [29]. The cells with a minimum of 500 genes expressed, total UMI counts > 1000, and the percentage of mitochondrial gene counts <20% were kept for subsequent analysis. UMI counts were normalized for each cell by the total expression, multiplied by 106, then log-transformed. We employed the Harmony algorithm to address batch effects, which is a robust and widely used method for integrating datasets across different experimental conditions. Specifically, we performed the following steps: (1) embedding the high-dimensional scRNA-seq data into a lower-dimensional space using principal component analysis (PCA). (2) Applying a soft clustering approach to group cells based on their expression. (3) For each cluster, we calculated batch-specific correction vectors and applied them to align the cells across different batches. (4) This process is repeated iteratively until convergence, resulting in a batch-corrected embedding.

Next, we discovered the variable genes by using the FindVariableFeatures function with parameters: selection.method = “vst”, nfeatures = 2000, and the ScaleData function was used to regress out variation due to differences in total UMIs per cell. Principal component analysis was used on the scaled expression profiles for the variable genes, and the top 30 principal components (PCs) were selected for subsequent analysis. Cells were clustered by using the FindClusters function. All cells were visualized by Uniform Manifold Approximation and Projection (UMAP) on the top 30 PCs. Cell types were assigned to each cluster by looking at the upregulated genes with the lowest Bonferroni-adjusted *p*-values. The cell–cell communication analysis was conducted by the R package cellchat (v1.6.1) [30]. To quantify the enrichment of cell subtypes in different groups, we adopted the method reported in the previous study to calculate the ratio of the observed and expected number of subtype cells in each group. The formula is as follows:

$$R_{o/e}=\frac{Observed}{Expected}$$

The observed value is the actual number of subtype cells in a specific tissue, and the expected value is obtained by counting the number of subtype cells and the number of specific tissues using the Chi-square test. When the *R*(*o*/*e*) > 1, this subtype is considered to be enriched in this group.

## 3. Results

### 3.1. Identification of a Prognostic Immune Gene Signature

**Figure 1:**
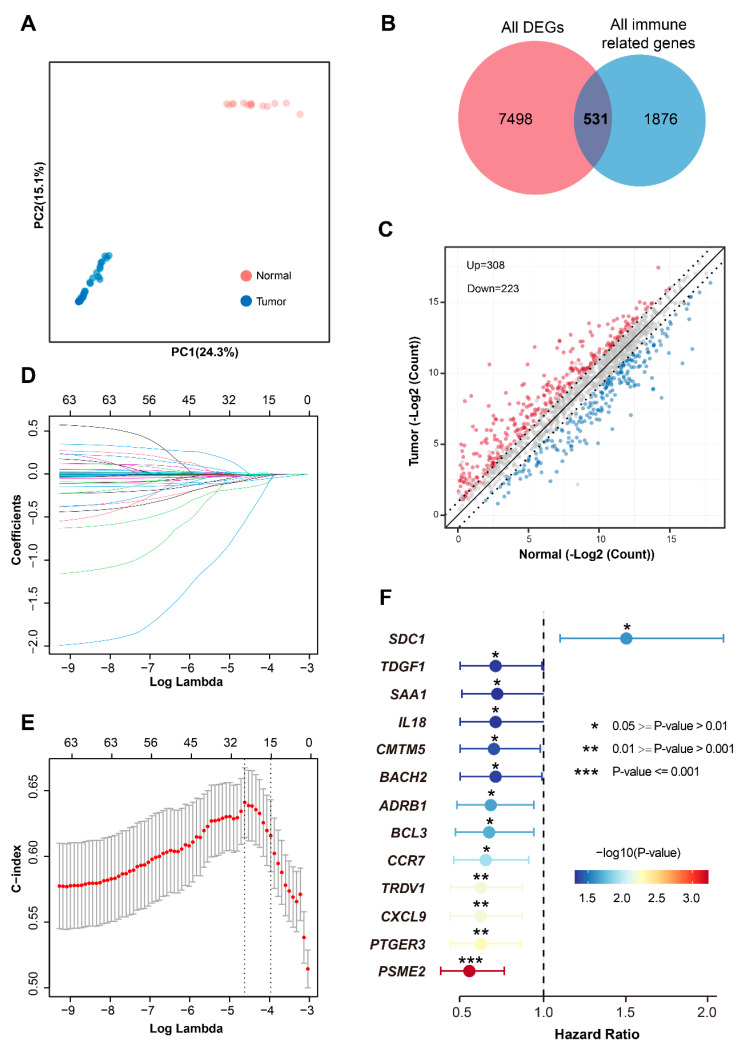
Development of the 13-gene immune prognostic signature. (**A**) PCA of transcriptomic profiles from 1075 breast tumors and 113 adjacent normal tissues (TCGA cohort). Red and blue dots represent normal and tumor samples, respectively. (**B**) Venn diagram identifying 531 breast cancer-associated immune-related DEGs through the intersection of 8029 DEGs and 2407 immune genes. (**C**) Volcano plot of DEGs. Red: 308 significantly upregulated genes; Blue: 223 downregulated genes. Gray dots: non-significant genes. (**D**) LASSO coefficient profiles of 63 candidate prognostic genes identified by univariate Cox regression. Each colored line represents the coefficient trajectory of one specific gene across varying levels of the L1 penalty. (**E**) Selection of optimal penalty parameter (lambda) via 10-fold cross-validation. (**F**) Forest plot of the final 13-gene signature showing hazard ratios (HR) and 95% confidence intervals. Colorful gradient represents the −log10 *p*-value of each gene. Abbreviations: PCA, principal component analysis; DEGs, differentially expressed genes; LASSO, least absolute shrinkage and selection operator; FC, fold change; *p*, *p*-value; λ, lambda; HR, hazard ratio; CI, confidence interval.

Leveraging transcriptomic profiles and clinical data from 1075 breast cancer tissues and 113 adjacent normal tissues in the TCGA database, we first assessed global expression differences. PCA revealed a significant divergence in the transcriptomic landscapes, demonstrating clear and distinct separation between tumor tissues and the adjacent normal counterparts (Figure 1A). Subsequent differential expression analysis identified 8029 significantly DEGs. Intersecting these DEGs with a comprehensive immunome gene set refined the focus to 531 breast cancer-associated immune-related DEGs, comprising 308 significantly upregulated and 223 significantly downregulated genes (Figure 1B,C; Table S3). To prioritize genes with clinical prognostic relevance, we performed univariate Cox proportional hazards regression analysis (*p* ≤ 0.05) on these immune-related DEGs, yielding 63 genes significantly associated with overall survival. To enhance prognostic model robustness, LASSO regression analysis was applied to further narrow the signature to 13 highly prognostic genes, establishing a refined immune-related prognostic gene signature (Figure 1D–F; Table S4). Additionally, several previous studies have characterized these genes in breast cancer. For example, *CXCL9*—a *Th1* chemokine that recruits CD8^+^ T cells—is recognized as a biomarker for “immunologically hot” tumors and predicts positive response to immune checkpoint inhibitors (e.g., pembrolizumab) in triple-negative breast cancer [15,31]. Moreover, *CCR7* facilitates lymph node metastasis by guiding tumor cell migration toward CCL21-expressing microenvironments and recruiting immunosuppressive Treg cells [16]. Individual patient risk scores are calculated using the following formula: Risk Score = (0.0009) × *SDC1* + (−0.0003) × *SAA1* + (−0.0576) × *TDGF1* + (−0.0726) × *BACH2* + (−0.0111) × *IL18* + (−0.0074) × *CMTM5* + (−0.0494) × *ADRB1* + (−0.0048) × *BCL3* + (0.0079) × *CCR7* + (−0.0003) × *CXCL9* + (−0.0077) × *PTGER3* + (−0.0174) × *TRDV1* + (−0.0074) × *PSME2*. This multivariate signature, derived through rigorous sequential analysis, encapsulates key immune mediators demonstrating significant collective prognostic value in breast cancer.

**Figure 2:**
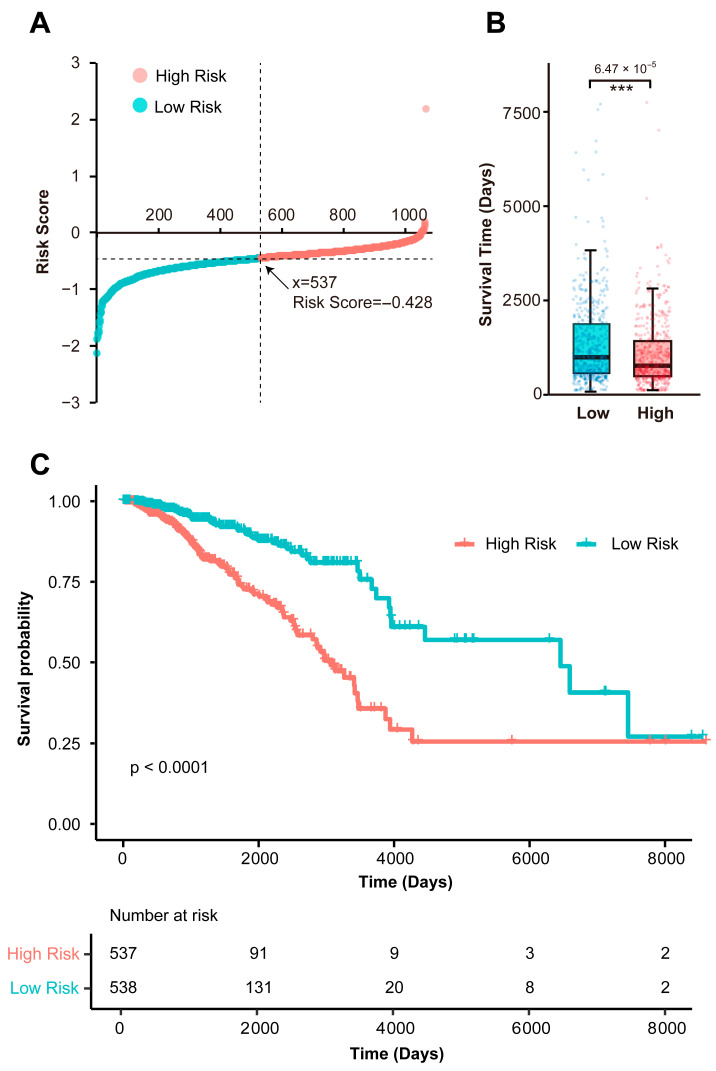
Prognostic performance of the signature in the TCGA cohort. (**A**) Risk score distribution and patient stratification (median cutoff: score = −0.428). Low-risk (blue, *n* = 537); High-risk (red, *n* = 538). (**B**) Comparative survival time analysis between risk groups. Significance levels: *** represents *p* < 0.0001. (**C**) Kaplan–Meier survival curves confirming significantly prolonged overall survival in low-risk patients. Abbreviations: TCGA, The Cancer Genome Atlas; *p*, *p*-value.

Following risk score calculation for each cancer sample, patients were stratified into low-risk (*n* = 537) and high-risk (*n* = 538) groups using the median risk score as the cutoff (Figure 2A). Comparative analysis of survival time revealed significantly prolonged survival in low-risk patients compared to high-risk counterparts (*t*-test: *p* = 6.47 × 10^−5^; Figure 2B), indicating the reliability of the signature to identify patients with divergent clinical trajectories. Consistent with this, Kaplan–Meier (KM) analysis demonstrated substantially higher survival probability in the low-risk group (log-rank *p* < 0.0001; Figure 2C), further validating the model’s discriminative power. Furthermore, the signature achieved a concordance index (C-index) of 0.678, indicating moderate-to-good prognostic accuracy for overall survival prediction.

To further assess performance across intrinsic molecular subtypes, we stratified the TCGA cohort into luminal A (*n* = 306), luminal B (*n* = 76), triple-negative breast cancer (TNBC, *n* = 98), and HER2+ (*n* = 35) subgroups. Risk scores were calculated using the established 13-gene coefficients, and optimal cutoffs were determined for each subtype using maximally selected rank statistics. Kaplan–Meier survival analyses demonstrated significant prognostic separation in luminal A (*p* = 0.0013), luminal B (*p* = 0.018), and TNBC (*p* = 0.004) subtypes, with the HER2+ group showing a non-significant trend (*p* = 0.13) (Figure S1). Although *p*-values were generally higher than in the unstratified cohort, these results suggest that the 13-gene signature may retain prognostic relevance across multiple molecular subtypes.

Taken together, we established and validated a clinically applicable 13-gene immune prognostic signature derived via Cox/LASSO-optimized transcriptomic profiling of 1075 breast tumors. The signatures demonstrated strong discriminative capacity, highlighting their potential as a standalone prognostic tool for breast cancer risk stratification.

### 3.2. Biological Characterization of the Immune-Related Gene Prognostic Signature

**Figure 3:**
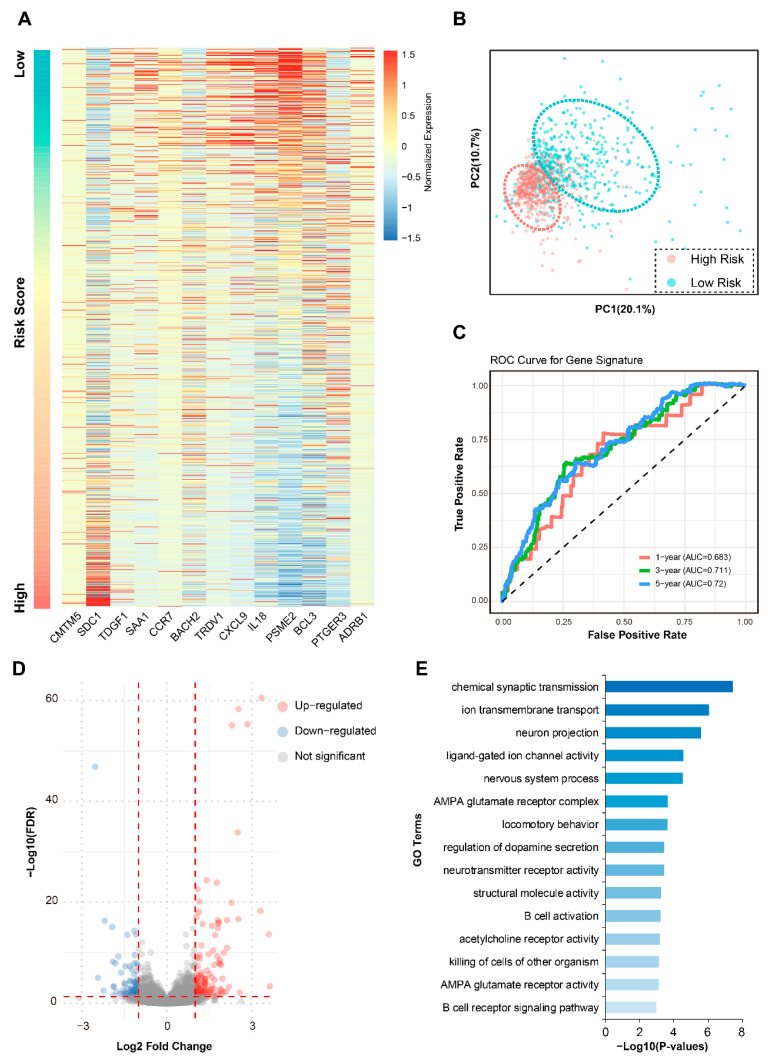
Characterization and comparison of 13 prognostic signatures between risk groups. (**A**) Heatmap of normalized expression of the 13 signature genes across patients ranked by ascending risk score. (**B**) PCA based on signature gene expression showing clear separation between risk groups. (**C**) Time-dependent ROC curves evaluating 1-, 3-, and 5-year overall survival prediction. (**D**) Volcano plot of DEGs between risk groups. (**E**) Bar diagram showing the top enriched Gene Ontology terms for DEGs. Abbreviations: TCGA, The Cancer Genome Atlas; PCA, principal component analysis; ROC, receiver operating characteristic; AUC, area under the curve; FDR, false discovery rate; GO, Gene Ontology; *p*, *p*-value.

We analyzed the expression patterns of these 13 prognostic genes in low-risk and high-risk patient groups. Heatmap visualization and PCA based solely on these signature genes revealed pronounced differential expression between the two risk-stratified tumor cohorts (Figure 3A,B). Notably, *IL18* and *PSME2* exhibited significantly higher expression levels in the low-risk group, whereas the high-risk group showed elevated expression of adverse genes, such as *SDC1*. This distinct expression dichotomy underscores the signature’s biological relevance and its intrinsic link to patient risk stratification. The prognostic performance of the signature was robustly validated using time-dependent ROC analysis. The AUC values for overall survival prediction reached 0.683 at 1 year, 0.711 at 3 years, and 0.72 at 5 years (Figure 3C), demonstrating moderate yet significant and temporally consistent predictive accuracy across short- and long-term clinical endpoints.

To elucidate global transcriptomic differences underlying the risk groups, we performed differential expression analysis comparing low-risk versus high-risk patients (using high-risk as control). This identified 570 significantly dysregulated genes, comprising 338 upregulated and 232 downregulated genes (Figure 3D). Gene Ontology (GO) enrichment analysis of these DEGs revealed significant overrepresentation in terms related to ‘chemical synaptic transmission’, ‘nervous system process’, ‘B cell activation’, and ‘killing of cells of other organism’ (Figure 3E), suggesting the potential involvement of these terms in shaping the aggressive phenotype associated with high-risk tumors.

### 3.3. Independent Validation of the Prognostic Signature in an External Cohort

**Figure 4:**
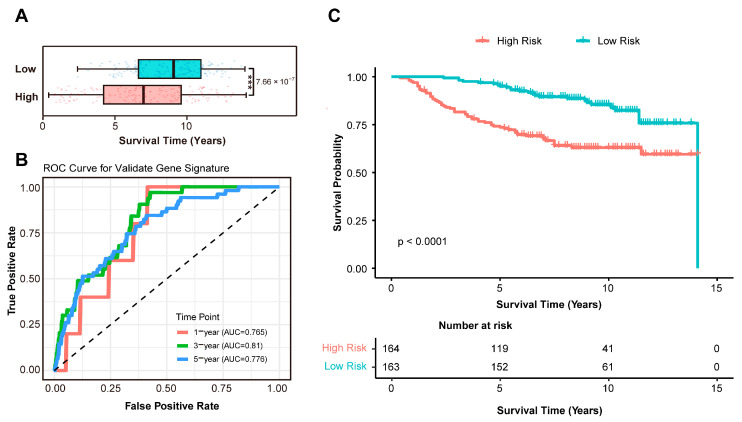
External validation in the GSE20685 cohort (*n* = 327). (**A**) Survival time comparison between risk groups in the validation cohorts. Significance levels: *** represents *p* < 0.0001. (**B**) ROC analysis for survival prediction in the validation cohorts. (**C**) Kaplan–Meier analysis confirming reproducible risk stratification in the validation cohorts. Abbreviations: *n*, number of patients; ROC, receiver operating characteristic; AUC, area under the curve; *p*, *p*-value.

To rigorously validate the clinical utility of the 13-gene prognostic signature, we applied it to an independent external cohort comprising 327 breast cancer samples. Using the established risk score formula, this validation cohort was stratified into 163 low-risk and 164 high-risk patients. Consistent with the training cohort, the low-risk group showed significantly prolonged overall survival time compared to the high-risk group (*t*-test: *p* = 7.66 × 10^−7^; Figure 4A). The signature’s predictive accuracy was further quantified using time-dependent ROC analysis, yielding AUC values of 0.765 at 1 year, 0.81 at 3 years, and 0.776 at 5 years, confirming its robust discriminatory power for both short- and long-term survival outcomes (Figure 4B). Concordantly, KM curves illustrated substantially higher survival probability in the low-risk group (log-rank *p* < 0.0001; Figure 4C). Together, these results unequivocally validate the 13-gene immune-related signature as a robust and transportable prognostic biomarker in breast cancer, capable of stratifying patients into clinically distinct risk groups with high reproducibility.

### 3.4. Differential Immune Microenvironment Landscapes Between Risk Groups

**Figure 5:**
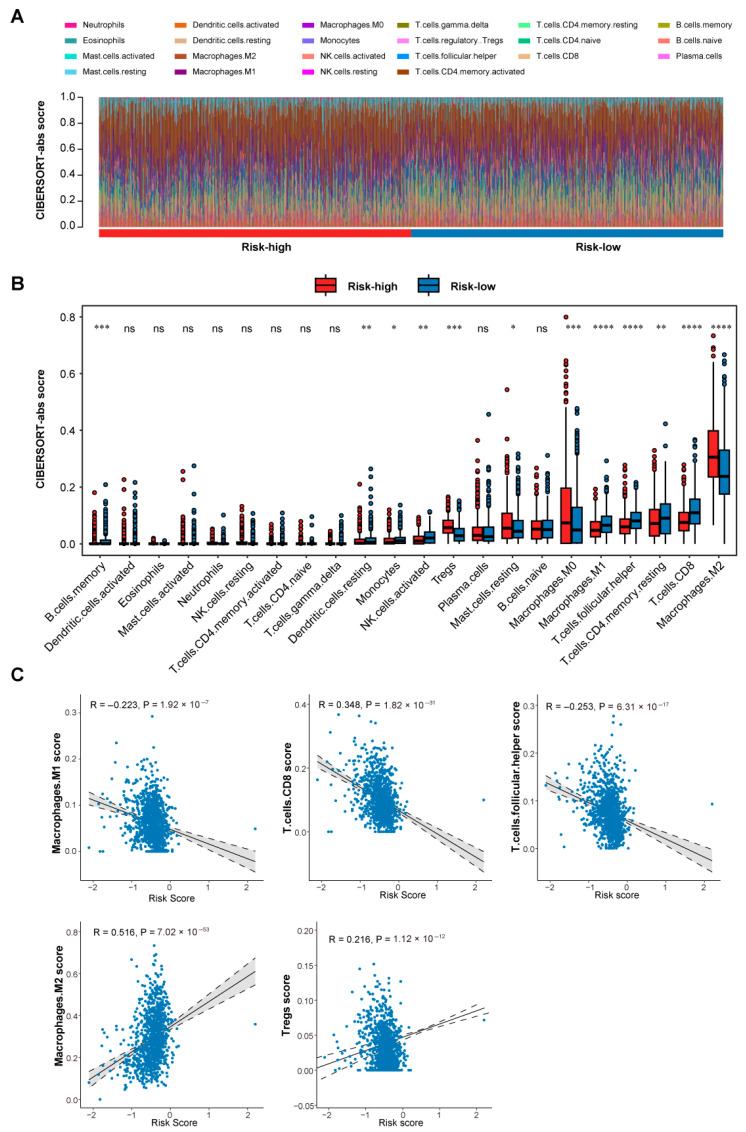
Immune microenvironment landscapes across risk groups. (**A**) Heatmap of CIBERSORT-inferred proportions of 22 immune cell types (rows) across patients. (**B**) Differential immune cell infiltration between groups. Significance levels: * represents *p* < 0.01, ** represents *p* < 0.001, *** represents *p* < 0.0001, **** represents *p* < 0.00001, ‘ns’ represents ‘not significant’ and *p* > 0.01. (**C**) Correlation analysis between risk score and immune subsets. Pro-inflammatory cells (CD8^+^ T cells, Tfh, M1) show negative correlations; immunosuppressive cells (Tregs, M2) show positive correlations. Analyses shown were conducted in the TCGA-BRCA cohort, with patients stratified into high- and low-risk groups based on the 13-gene signature. Abbreviations: CIBERSORT, Cell-type Identification By Estimating Relative Subsets Of RNA Transcripts; TCGA, The Cancer Genome Atlas; Tregs, regulatory T cells; *p*, *p*-value; ns, not significant.

We further characterized the differences in immune microenvironment between low-risk and high-risk patient groups in the TCGA-BRCA cohort. Using the CIBERSORT-abs algorithm, we estimated the proportions of 22 distinct immune cell populations for each patient in each group (Figure 5A). Strikingly, low-risk patients exhibited significantly higher proportions of M1-like macrophages, CD8^+^ T cells, and T follicular helper (Tfh) cells compared to the high-risk group (Wilcoxon test, *p*-value < 0.0001; Figure 5B). Conversely, the high-risk group was characterized by significantly elevated proportions of M2-like macrophages and Tregs (Wilcoxon test, *p*-value < 0.0001). These patterns were reinforced by correlation analysis between these key immune cell subsets and the established risk score. The CIBERSORT scores of pro-inflammatory/effector cells (M1-like macrophages: R = −0.223, *p* = 1.92 × 10^−13^; CD8^+^ T cells: R = −0.348, *p* = 1.82 × 10^−31^; Tfh cells: R = −0.253, *p* = 6.31 × 10^−17^) were significantly inversely correlated with the risk score (Figure 5C). In contrast, the scores of immunosuppressive cells (M2-like macrophages: R = 0.516, *p* = 7.02 × 10^−53^; Tregs: R = 0.216, *p* = 1.12 × 10^−12^) showed significant positive correlations with the risk score. Collectively, these results define contrasting immune landscapes. Low-risk patients display an antitumor, pro-inflammatory microenvironment enriched with cytotoxic effectors (CD8^+^ T cells), classically activated macrophages (M1-like), and T cells supporting adaptive immunity (Tfh). Conversely, high-risk patients exhibit a profoundly immunosuppressive, pro-tumorigenic microenvironment dominated by alternatively activated macrophages (M2-like) and Tregs, which actively suppress immune responses and promote tumor progression.

### 3.5. Single-Cell Dissection of Risk-Associated Microenvironments

**Figure 6:**
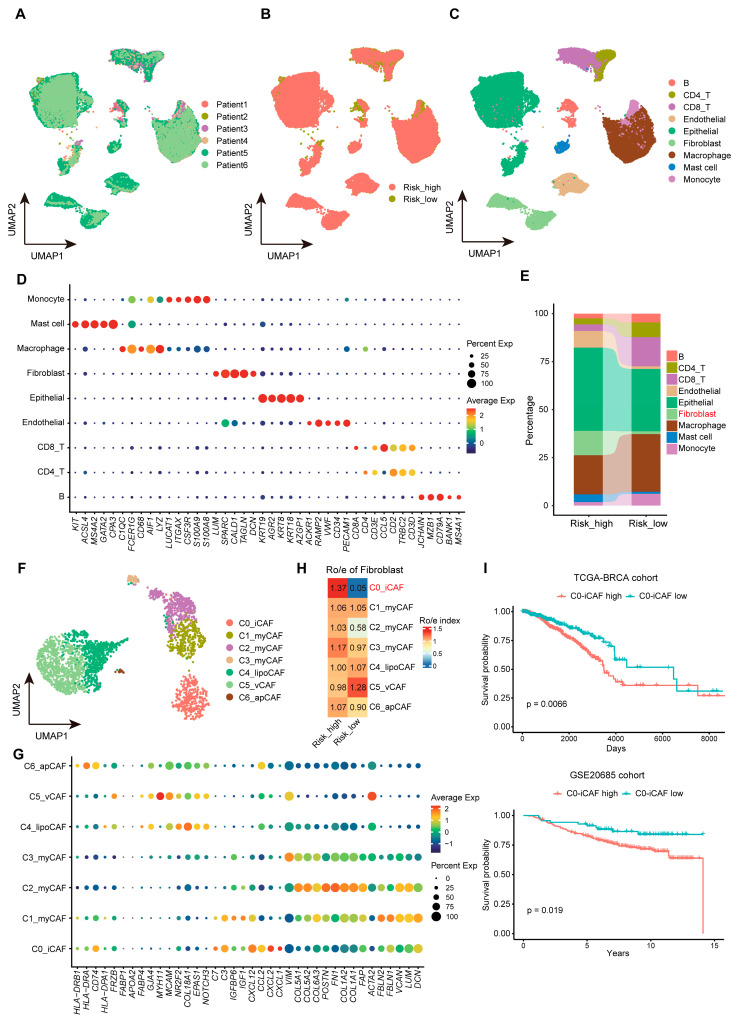
Single-cell dissection of risk-associated microenvironments. (**A**,**B**) UMAP visualization of single cells from six breast cancer samples after batch correction, distinguishing the high- and low-risk groups. (**C**,**D**) Unsupervised clustering identifying 9 major cell types using canonical markers (**C**), and marker gene expressions in major cell types (**D**). (**E**) Stacked bar chart showing the differential cell type proportion. High-risk tumors show expanded epithelial cells and fibroblasts. (**F**,**G**) Fibroblast sub-clustering revealing five CAF subtypes: inflammatory (iCAF), myofibroblastic (myCAF), lipid-associated (lipoCAF), vascular (vCAF), and antigen-presenting (apCAF). (**H**) Ro/e analysis of CAFs between high- and low-risk groups, exclusive enrichment of iCAFs in high-risk tumors. (**I**) Survival analysis validating iCAF enrichment score as a poor-prognosis indicator. Abbreviations: scRNA-seq, single-cell RNA sequencing; UMAP, uniform manifold approximation and projection; CAF, cancer-associated fibroblast; iCAF, inflammatory CAF; myCAF, myofibroblastic CAF; apoCAF, antigen-presenting CAF; TCGA, The Cancer Genome Atlas; *p*, *p*-value.

To dissect the cellular basis underlying the immunosuppressive microenvironment observed in high-risk patients (Figure 5), we interrogated a publicly available single-cell RNA-seq dataset of breast cancer (GSE254991, *n* = 6). After calculating risk scores via pseudobulk aggregation, these samples were stratified into high- and low-risk cohorts (*n* = 3 per group). Following batch correction and dimensionality reduction (Figure 6A,B), unsupervised clustering identified nine major cell types: B cells, CD4^+^ T cells, CD8^+^ T cells, endothelial cells, epithelial (tumor) cells, fibroblasts, macrophages, mast cells, and monocytes (Figure 6C,D). Interestingly, comparative analysis uncovered significant expansion of epithelial cells and CAFs in the high-risk samples (Figure 6E), suggesting an activated stromal compartment and epithelial remodeling consistent with the immunosuppressive phenotype of the high-risk group.

Given the known heterogeneity of CAFs [32,33], we further subclassified fibroblasts into five functionally distinct subsets based on canonical markers, including iCAFs, myofibroblastic CAFs (myCAFs), lipid-associated CAFs (lipoCAFs), vascular CAFs (vCAFs), and antigen-presenting CAFs (apCAFs) (Figure 6F,G; Table S5). Strikingly, iCAFs were exclusively enriched in high-risk tumors while nearly absent in low-risk counterparts (Figure 6H), implicating iCAF infiltration as a key mediator of adverse outcomes. Moreover, to clinically validate this association, we identified C0-iCAF-specific signature genes and performed gene set variation analysis (GSVA) in training and validation cohorts. Patients with high C0-iCAF scores exhibited significantly worse overall survival in both datasets (*p* = 0.0066 for TCGA cohorts, and *p* = 0.019 for validation cohorts; Figure 6I), indicating that iCAF-driven pro-inflammatory remodeling may constitute a conserved mechanism underlying poor prognosis in high-risk patients. Collectively, these findings mechanistically link the 13-gene signature to iCAF-driven immunosuppression, providing a cellular rationale for the adverse prognosis in high-risk patients.

## 4. Discussion

This study establishes a rigorously validated 13-gene immune signature that effectively stratifies breast cancer patients into distinct prognostic subgroups. The genes included in this signature are broadly involved in immune regulation, antigen processing, cytokine signaling, and tumor microenvironment remodeling. Critically, this signature’s clinical utility is underscored by its high discriminatory power (C-index = 0.678–0.703) and consistent predictive accuracy across both short- and long-term survival endpoints (5-year AUC = 0.72; external 3-year AUC = 0.81). Several components of the signature have been implicated in antitumor immunity. For example, *CXCL9* has been implicated in shaping the immune landscape of breast cancer. Elevated *CXCL9* expression correlates with increased infiltration of CD8^+^ T cells and other effector immune populations, reflecting a more immunologically active tumor status. In breast cancer, higher *CXCL9* levels are generally linked to a favorable prognosis and enhanced antitumor immunity, including potential responsiveness to immunotherapies, particularly in ER-negative and triple-negative subtypes [34,35,36]. IL-18 is known to stimulate IFN-γ production and enhance NK/T cell cytotoxicity in immunological models, and elevated serum IL-18 has been associated with prognosis in breast cancer patients [37,38]. In contrast, *CCR7* has been reported in breast cancer meta-analyses to correlate with worse survival and lymph node metastasis [39]. While direct mechanistic evidence for *CCR7* recruiting Tregs in breast cancer is limited, *CCR7*’s role in lymphatic migration and immune cell homing suggests a plausible link to immunosuppression.

Comprehensive immune deconvolution revealed strikingly divergent immune infiltrates between risk groups. Low-risk tumors were characterized by abundant M1-like macrophages, CD8^+^ T cells, and Tfh cells, constituting a pro-inflammatory, antitumor milieu. In contrast, high-risk tumors displayed increased infiltration of M2-like macrophages and regulatory T cells, indicative of a profoundly immunosuppressive environment. These findings are consistent with previous reports that M1 macrophages and CD8^+^ T cells predict better immunotherapeutic outcomes, whereas M2 macrophages and Tregs promote tumor growth and immune escape [40]. The significant correlations between the risk score and immune cell proportions reinforce that our signature functionally captures immune polarization within the TME, providing both prognostic and biological interpretability.

Single-cell transcriptomic analysis provided critical insight into the cellular architecture underlying the prognostic stratification derived from our immune gene signature. In particular, high-risk tumors displayed substantial expansion of CAFs and malignant epithelial cells, indicative of active stromal remodeling and epithelial–mesenchymal interactions that promote immune evasion. CAFs are activated stromal cells within the tumor microenvironment (TME), characterized by spindle-like morphology, α-SMA/FAP expression, and secretion of pro-tumorigenic factors (e.g., IL-6, CXCL12, TGF-β), originating from diverse sources including resident fibroblasts, mesenchymal stem cells, and transdifferentiated endothelial cells, with high functional plasticity driving TME remodeling [41,42]. Extensive prior studies have established CAFs as central orchestrators of immunosuppression and tumor progression [43,44,45,46,47]. Accordingly, we assumed that the expansion of CAFs could majorly explain the aggressive phenotypes in the high-risk patients. Importantly, our CAF subclassification analysis revealed a predominant enrichment of iCAFs in high-risk tumors, whereas their presence was minimal in low-risk cases. iCAFs represent a transcriptionally distinct fibroblast subset characterized by the secretion of pro-inflammatory cytokines (such as *IL6*, *LIF*, and *CXCL12*) and chemokines that modulate immune cell trafficking and polarization [48]. The spatial enrichment of iCAFs in high-risk tumors may establish a self-reinforcing immunosuppressive circuit: iCAF-derived cytokines recruit Tregs and polarize M2 macrophages, which reciprocally secrete TGF-β and PD-L1 to further activate iCAFs and inhibit cytotoxic T cells, creating a feed-forward loop that potentially drives aggressive phenotypes. Patients with high iCAF enrichment scores exhibited significantly worse survival across independent cohorts, underscoring the robustness and clinical applicability of our 13-gene immune signature. These findings suggest that the adverse prognosis of high-risk patients may be attributed, at least in part, to iCAF-driven pro-inflammatory remodeling and immunosuppression within the tumor microenvironment. Targeting the fibroblast–immune cell axis therefore represents a promising therapeutic strategy to counteract stromal-mediated immune tolerance. The iCAF-dominated immunosuppressive niche in our high-risk cohort resonates with pathway dysregulations identified in chemotherapy-resistant tumors. Li et al. reported that *CRTC1* mutations and Signature 3 (homologous recombination deficiency) are enriched in chemotherapy-resistant breast cancer, activating JAK-STAT signaling to promote immune evasion [49]. Our single-cell data analysis extends this by revealing iCAFs as central orchestrators of such dysfunction: they secrete IL-6/LIF to polarize macrophages toward M2 states while recruiting Tregs via CXCL12—mirroring PPY-induced iCAF mechanisms in pancreatic cancer that suppress CD8^+^ T cells through EGFR/NF-κB [20,50]. These findings nominate iCAF-targeted co-therapies as essential for reversing treatment resistance. Notably, mTOR inhibitors (e.g., Temsirolimus) show high sensitivity in iTILs-low tumors [51], suggesting synergy with our signature-guided interventions.

Recent advances highlight the transformative potential of multimodal data fusion in refining risk stratification. For instance, Qian et al. developed BMU-Net—a Transformer-based model that integrates mammography, ultrasound, and clinical data—achieving 90.1% accuracy in prospective validation, surpassing radiologists in pathological grading tasks [52]. Similarly, transformer architectures have demonstrated an AUC of 0.943 for cancer detection and 0.826 for 5-year risk prediction by leveraging longitudinal imaging data [53]. These studies underscore that combining molecular signatures with imaging and clinical variables can overcome limitations of unimodal approaches, a direction critical for translating our immune signature into clinical practice. Moreover, our signature’s performance parallels that of glycogene-based models, such as the 19-glycogene signature identified by Lin et al., which also linked low-risk status to enhanced CD8^+^ T-cell infiltration and improved immunotherapy efficacy [54]. Additionally, our LASSO-Cox framework demonstrates competitive performance (C-index 0.703 externally), but emerging machine learning algorithms offer avenues for enhancement. The CoxBoost + RSF model developed by Wang et al.—which integrates 19 iTILs-related genes through 101 algorithm combinations—achieved an exceptional 5-year AUC of 0.959, outperforming 38 existing models [51]. Such approaches could refine our signature by capturing nonlinear interactions between immune mediators. For survival analysis, AFT frailty models with LASSO regularization recently demonstrated superior handling of unobserved heterogeneity (frailty variance 0.42, C-index 0.882) [55]. These methodological innovations, combined with explainable AI techniques like SHAP value analysis (which identified liver metastases and QoL scores as key survival predictors in advanced disease) [56], provide frameworks to interpret complex feature contributions in our risk stratification system.

Despite robust validation, limitations warrant consideration. In our subtype-specific analyses, the 13-gene immune signature showed prognostic separation in luminal A, luminal B, and TNBC subtypes, while the HER2+ group exhibited only a non-significant trend (Figure S1). We attribute this, at least in part, to the limited sample sizes and event counts within certain subtypes, particularly the HER2+ cohort (*n* = 35), which likely reduced statistical power. Larger, subtype-enriched datasets will be essential for more robust evaluation and potential refinement of the signature for specific molecular contexts. Our signature’s performance in HER2+ subtypes requires further evaluation, particularly given emerging evidence that functional BRCA2 loss-of-function variants (classified via CRISPR-Cas9 saturation editing) confer differential therapeutic vulnerabilities [57]. Future studies should integrate germline genetic risk with microenvironmental signatures to enable comprehensive risk assessment. Moreover, prospective trials are needed to validate ctDNA-guided intervention based on our risk groups, similar to ongoing efforts in neoadjuvant settings where Zhang et al. achieved high pathological response rates by combining immune checkpoint blockade with anti-angiogenic agents [11]. In addition, future work could benefit from adopting the integrative strategy reported in the recent study [58], which combined large-scale transcriptomic profiling with high-resolution single-cell analyses to refine immune-related prognostic models. Applying a similar approach in the context of our 13-gene immune signature may enable more precise dissection of cell-type–specific contributions to the tumor microenvironment, and facilitate identification of novel interaction networks between inflammatory CAFs and immune cell subsets. Such integration of bulk and single-cell datasets, potentially coupled with spatial transcriptomics, could enhance both the biological interpretability and predictive power of the signature, accelerating its translation into clinical decision-making. Notably, spatial multi-omics studies have identified conserved CAF subtypes with defined positional preferences and clear associations with immune cell neighborhoods, suggesting that the localization of iCAFs in specific niches (e.g., perivascular or dense stromal regions) may contribute to localized immunosuppressive gradients that shape immune cell distribution and function [59]. Incorporating spatial transcriptomic data in future work will therefore be essential for defining the spatially resolved roles of iCAFs in the tumor microenvironment. Additionally, functional confirmation, such as immunohistochemistry to visualize iCAF localization, co-culture assays to assess their effect on immune cell activity, or in vivo models to test causal roles, would strengthen the mechanistic interpretation of our findings. Future studies incorporating these approaches will be critical to corroborate the iCAF-mediated immune suppression observed in our high-risk cohort and to refine the translational applicability of our 13-gene immune signature for therapeutic intervention.

Moreover, both the TCGA and validation cohorts are largely based on publicly available datasets, which may limit the representation of diverse ethnicities and clinical backgrounds. This potential sampling bias could affect the generalizability of our prognostic signature. Future studies will be necessary to validate and refine the model in larger, prospectively collected, and demographically heterogeneous cohorts. Furthermore, with the rapid development of deep learning and artificial intelligence (AI) in biomedical research, future extensions of our work could leverage advanced network-based approaches to improve prognostic accuracy. For example, convolutional neural network (CNN)-driven subspace clustering methods, as demonstrated in recent work [60], could be applied to integrate multi-omics data and capture complex nonlinear patterns within the tumor microenvironment. Such integration may help uncover novel molecular subgroups and enhance the clinical applicability of our prognostic model. Although our 13-gene immune signature demonstrated robust prognostic performance in retrospective cohorts, direct head-to-head comparison with established commercial assays such as Oncotype DX and MammaPrint is not possible within the current datasets. Commercial assays largely assess proliferation and hormone-related pathways, while our model emphasizes immune regulation and tumor microenvironment remodeling, particularly iCAF-associated immunosuppression, thereby providing potentially complementary prognostic information. In terms of clinical applicability, translation of this model into routine practice will require further evaluation of its feasibility—including assay standardization, platform compatibility, and turnaround time—within real-world clinical workflows. Moreover, before clinical implementation, the model must be validated in large, multi-center prospective cohorts (typically several hundred patients with standardized follow-up) to confirm its reproducibility, clinical utility, and performance across different molecular subtypes.

## 5. Conclusions

Based on integrated transcriptomic analysis of 1075 breast cancer patients and independent validation, we established and rigorously validated a 13-gene immune prognostic signature that robustly stratifies patients into distinct risk groups with divergent survival outcomes. This signature is mechanistically anchored in polarized tumor immune microenvironments: low-risk patients exhibit an antitumor milieu enriched with cytotoxic CD8^+^ T cells, Tfh cells, and M1 macrophages, while high-risk patients display an immunosuppressive phenotype showing higher proportions of Tregs and M2 macrophages. Crucially, single-cell RNA sequencing revealed iCAFs as pivotal drivers of the high-risk immunosuppressive niche, with iCAF enrichment clinically linked to adverse prognosis. This study delivers a clinically translatable prognostic tool and nominates iCAF-targeted strategies as a promising therapeutic avenue for aggressive breast cancer.

## Data Availability

The transcriptomic datasets (RNA-seq) and corresponding clinical data from TCGA (TCGA-BRCA, *n* = 1075 tumors and 113 normals) are publicly accessible via the Genomic Data Commons (GDC) portal (https://portal.gdc.cancer.gov/, accessed on 20 March 2023). The independent validation dataset (GSE20685, *n* = 327) is available at the GEO repository (https://www.ncbi.nlm.nih.gov/geo/, accessed on 20 March 2023). Single-cell RNA-seq data analyzed in this study were sourced from GEO accession GSE254991.

## Supplementary Materials

The following supporting information can be downloaded at https://www.mdpi.com/article/10.3390/biomedicines13122966/s1. Figure S1: Prognostic performance of the 13-gene immune signature across different molecular subtypes of breast cancer in the TCGA cohort; Table S1: Clinical data of 1075 tumor samples from TCGA database; Table S2: Clinical data of 327 validation cohorts; Table S3: 531 Differentially Expressed Immune-related Genes; Table S4: Annotation of the 13 Genes in the Risk Score Model; Table S5: Up-regulated genes in the C0_iCAF.
