# Supplementary material for: An Immune Gene Signature Stratifies Breast Cancer Prognosis Through iCAF-Driven Immunosuppressive Microenvironment
Source: Biomedicines. 2025 Dec 2;13(12):2966. doi: 10.3390/biomedicines13122966 (PMC12730545; doi:10.3390/biomedicines13122966)
Supplement: Supplementary file 1 [file biomedicines-13-02966-s001.zip › biomedicines-3946031.Supplementary Figure.pdf]

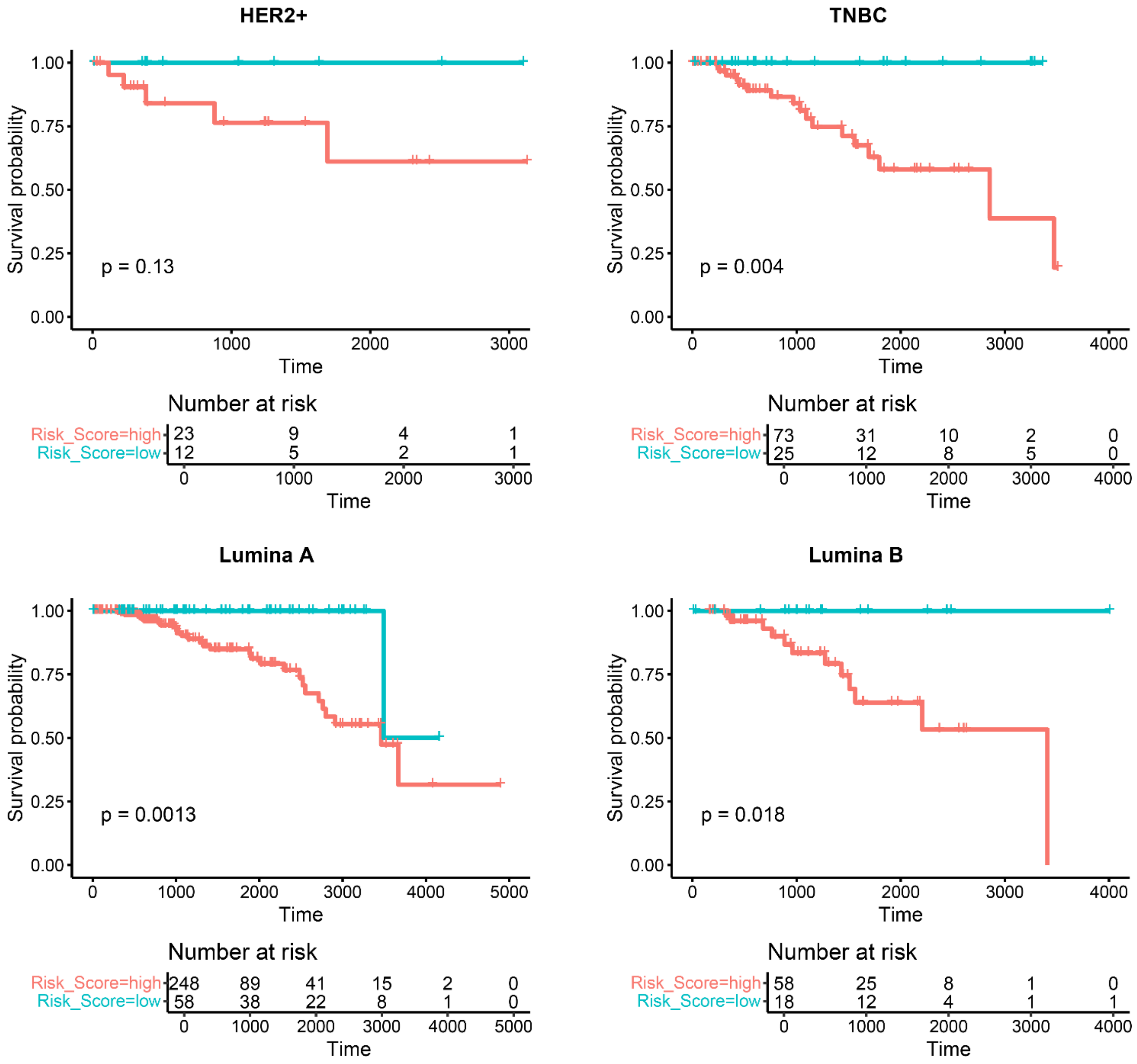

Figure S1: Prognostic performance of the 13-gene immune signature across different molecular subtypes of breast cancer in the TCGA cohort. TCGA breast cancer cases were stratified into luminal A ($n = 306$), luminal B ($n = 76$), triple-negative breast cancer (TNBC; $n = 98$), and HER2+ ($n = 35$) subtypes based on clinical annotation. Risk scores were calculated using the established 13-gene coefficients, and optimal cutoffs for high- and low-risk groups were determined for each subtype using maximally selected rank statistics. Log-rank *p*-values: luminal A, 0.0013; luminal B, 0.018; TNBC, 0.004; HER2+, 0.13.
